# Access to tablet injectable opioid agonist therapy in rural and smaller urban settings in British Columbia, Canada: a qualitative study

**DOI:** 10.1186/s13011-023-00525-2

**Published:** 2023-03-03

**Authors:** Geoff Bardwell, Jeanette M. Bowles, Manal Mansoor, Dan Werb, Thomas Kerr

**Affiliations:** 1 School of Public Health Sciences, Faculty of Health, University of Waterloo, 200 University Avenue West, Waterloo, ON N2L 3G1 Canada; 2 British Columbia Centre On Substance Use, 400-1045 Howe Street, Vancouver, BC V6Z 2A9 Canada; 3 Department of Medicine, University of British Columbia, St. Paul’s Hospital, 608-1081 Burrard Street, , Vancouver, BC V6Z 1Y6 Canada; 4 Centre On Drug Policy Evaluation, MAP Centre for Urban Health Solutions, St. Michael’s Hospital, 209 Victoria St, Toronto, ON M5B 1T8 Canada; 5 Division of Infectious Diseases & Global Public Health, University of California San Diego, 9500 Gilman Drive, La Jolla, CA 92023 USA

**Keywords:** Rural health, Drug use, Tablet injectable opioid agonist therapy, Hydromorphone, Overdose prevention, Qualitative interviews, Rural and smaller urban Canada

## Abstract

**Background:**

Rural and smaller urban settings in Canada are disproportionately impacted by the overdose crisis, highlighting the need for novel public health interventions within these jurisdictions. Tablet injectable opioid agonist therapy (TiOAT) programs have been implemented in select rural communities as a means to address drug-related harms. However, little is known about the accessibility of these novel programs. Therefore, we conducted this study to understand the rural context and factors that affected access of TiOAT programs.

**Methods:**

Between October 2021 to April 2022, individual qualitative semi-structured interviews were conducted with 32 individuals enrolled in a TiOAT program at participating rural and smaller urban sites in British Columbia, Canada. Interview transcripts were coded using NVivo 12 and data were analyzed thematically.

**Results:**

TiOAT access varied considerably. TiOAT delivery in rural settings is complicated due to geographic challenges. Participants who were homeless and staying at a nearby shelter or those in centrally-located supportive housing had minimal issues compared to those living in more affordable housing on the outskirts of town with limited transportation options. Dispensing policies that required daily-witnessed ingestion multiple times daily were challenging for most. Only one site provided evening take-home doses whereas participants at the other site could only resort to the illicit opioid supply to address withdrawal outside of program hours. Participants described the clinics as providing a positive and familial social environment compared to experiences of stigma elsewhere. Medication interruptions did occur when participants were in hospital and custodial settings, leading to withdrawal, program discontinuation, and overdose risk.

**Conclusions:**

This study highlights the beneficial ways in which health services tailored for people who use drugs can create a stigma-free environment with an emphasis on social bonds. Other factors such as transportation access, dispensing policies, and access in rural hospitals and custodial settings produced unique challenges for rural people who use drugs. Public health authorities in rural and smaller settings should consider these factors when designing, implementing, and scaling up future substance use services, including TiOAT programs.

## Background

Since 2016, communities across North America have been enduring an overdose epidemic that has been exacerbated by the highly toxic and unregulated illicit drug supply and, more recently, the COVID-19 pandemic [1–3]. In the Canadian province of British Columbia (BC), for example, the drug toxicity death rate has increased from 20.5 deaths per 100,000 individuals in 2016 to 41.6 deaths per 100,000 individuals in 2022 [4]. Public health interventions intended to address these preventable deaths, including supervised consumption services, novel opioid agonist therapy programs, and safer opioid supply programs, have been inconsistently implemented across Canada with current coverage failing to achieve meaningful population-level reductions in overdose mortality [5–7].

In the current era where the unregulated opioid supply is contaminated with several adulterants (e.g., fentanyl, carfentanil, xylazine, benzodiazepines), there is a need for novel approaches to prevent overdose, including alternatives to first-line opioid agonist therapies (OAT; e.g., methadone, buprenorphine). While these medications have been successful for many individuals and continue to be considered the gold standard of treatment among physicians, program retention rates in BC continue to be low [8]. Novel OAT programs therefore need to be expanded and evaluated, including injectable OAT options [9]. For individuals who have not benefited from first-line OAT, injectable OAT options have more recently emerged in select jurisdictions across Canada, and there now exists national clinical guidelines for injectable OAT [10]. Recommended medications include diacetylmorphine and hydromorphone [10] – though the latter has been more widely available at existing injectable OAT programs in Canada [11].

Research on injectable OAT is not a new phenomenon [12]. Indeed, the use of diacetylmorphine to treat opioid use disorder has been studied in both Europe and Canada. In the European context, studies have been conducted in Switzerland, Spain, Germany, and the Netherlands, and have demonstrated high adherence and retention rates (e.g., 70 to 94% at 12-months), among other positive health and social outcomes [13–16]. For example, in a randomized controlled trial in Spain, it was reported that patients on injectable diacetylmorphine had greater improvements in physical health, reductions in illicit heroin use, and involvement in criminalized activities compared to the patients on oral methadone [15]. In the Canadian context, similar research has been conducted on the use of both diacetylmorphine and liquid hydromorphone for the treatment of severe opioid use disorder and studies have found injectable diacetylmorphine to be more effective than oral methadone, and that when comparing diacetylmorphine with liquid hydromorphone, hydromorphone is noninferior and should be explored as a treatment option [9, 17, 18].

There is very limited research on TiOAT specifically, and no known studies have compared liquid hydromorphone to tablet formulations. Amidst an overdose epidemic, TiOAT initially started as a pilot project in 2019 in Vancouver as a harm reduction intervention for individuals with severe opioid use disorder who were actively injecting unregulated opioids [19]. Existing research on TiOAT is limited to environmental scans on the expansion of multiple novel OAT programs (including both liquid and tablet hydromorphone) as well as safer opioid supply prescribing across Canada during the COVID-19 pandemic [11, 20, 21]. These environmental scans are primarily descriptive and utilize surveys and qualitative interviews with service providers only, and while there is a mixed-methods provincial evaluation underway [22], there are no known publications as of yet. Despite promising results from pre-overdose epidemic clinical trials on liquid hydromorphone specifically [9, 17, 18], there has been limited research during the current overdose crisis that has examined the effectiveness of these novel programs during the fentanyl era as well as any programmatic access barriers and facilitators, and recent Canadian studies on injectable OAT (including tablet and liquid formulations) have occurred exclusively in large urban centres [23–25].

While the overdose epidemic in BC is impacting all jurisdictions across the province, rural and smaller urban communities are disproportionately impacted [26]. For example, nine out of the top ten Local Health Areas with the highest overdose mortality rates are in rural and smaller urban settings [4]. Despite this fact, most Canadian research examining drug use, overdose risk, and related public health interventions occurs in large cities and gaps remain in our understanding of the factors that may affect OAT program access, adherence, and retention, including novel injectable OAT, in rural settings and smaller cities [27].

In 2020 and 2021, two programs that provide TiOAT (i.e., hydromorphone) were launched in two rural and smaller urban settings in BC. These programs exist in Duncan (rural; population ~ 5,000), which is located on Vancouver Island, and in Kamloops (rural/small urban; population ~ 98,000), which is located in the province’s interior — a 4 h drive from Vancouver, the province’s largest urban centre. The Kamloops program has two sites whereas the Duncan program has one site. These programs provide TiOAT in clinical settings. Despite the potential promise of these programs in addressing overdose risk and other drug-related harms, to our knowledge no research currently exists on how these programs operate in rural and smaller settings. Therefore, we sought to understand the rural contextual factors that affect TiOAT program access among program participants.

## Methods

### Methodology

We merged qualitative research methodologies – case study and narrative inquiry [28]. Case studies have a predefined boundary (nature, temporality, scope) and focus on a relevant social group and/or geographical location [29]. We specifically utilized an intrinsic case study, which focuses on the uniqueness of a phenomenon (i.e., TiOAT programs in rural/smaller urban settings) rather than a collective case study, which focuses on multiple cases to produce an expansive understanding of phenomena [29]. Case studies provide insights into how a health service may be accessed by individuals, including any barriers or facilitators in its delivery [29], making this methodological approach appropriate for a study on TiOAT access. While case study provides the context of a particular phenomenon, narrative inquiry allows us to understand how participants situate their day-to-day experiences in that particular context and utilizing open-ended questions allows participants to share their stories [28, 30].

### Study sites

Participants were recruited from three TiOAT program sites across two rural and smaller urban communities. We utilized criteria from BC Ministry of Health in characterizing these communities as rural and smaller urban, which considers population size and proximity to larger population centres and health services in defining rurality [31]. BC Ministry of Health provided funding to specifically evaluate these three program sites. In Kamloops there are two TiOAT clinics, both of which are run by the same social service provider. One is located on the “Southshore” embedded in a pharmacy in the downtown area and the other is on the “Northshore” – an area considered to be more low-income – and is nested in a larger social service building with an embedded supervised consumption service, harm reduction supply access, and other services targeted toward people who use drugs. In Duncan there is only one clinic and it is centrally-located in a social service that also provides supervised consumption (including inhalation), harm reduction supplies, substance use services, referrals to health services, and other social supports. Program participants were required to attend the clinics multiple times per day (n = 5) to get their full dosage. Participants are provided 1 to 2 8 mg hydromorphone tablets per visit. Participants had the option to consume their medication orally, intranasally, intravenously, or intramuscularly, all under the supervision of nursing staff. Those at the Duncan site were provided with take-away doses (unsupervised) in the evening whereas those in the Kamloops program did not receive take-away doses.

### Recruitment

When we started recruiting participants, the Kamloops program had been open since February 2020 whereas the Duncan program had been open since September 2021. Interviews across both sites occurred between October 2021 to April 2022. To be eligible, participants had to be 19 years of age or older, have been enrolled in the local TiOAT program for at least 1-month, and live in Kamloops or Duncan or their surrounding areas. This allowed us to recruit participants that were currently enrolled in the program as well as those who have discontinued enrollment (after utilizing the program for at least 1-month). Posters were displayed in the TiOAT clinical settings and dedicated on-site staff assisted with recruitment and screening. Staff were directed to refer all participants who met inclusion criteria. Given that recruitment occurred in clinics that were integrated among other services, this allowed staff to refer individuals who may be accessing other services (e.g., harm reduction supplies) but not the TiOAT clinic, including those who may have discontinued or disengaged from the programs. Interested participants were provided with informed consent forms and signed up for remote interviews. At the time of data collection there were approximately 30 TiOAT recipients in Kamloops and 10 in Duncan and our recruitment numbers reflected these differences (Kamloops participants, *n* = 25; Duncan participants, *n* = 7).

### Interviews

Thirty-two interviews were conducted by GB (Principal Investigator) and MM (research coordinator). Due to COVID-19 restrictions, initial interviews were conducted via video communications software and participants were situated in a private offices at the TiOAT clinics. Additional interviews in Kamloops were conducted in-person at a private office and interviewers followed pandemic safety protocols. Qualitative semi-structured open-ended interviews were conducted with participants who were each provided with a $30 cash honorarium [32]. An interview guide was developed with feedback from a provincial community advisory board comprised of people with lived experience of drug use and engagement in OAT programs to ensure relevancy and comprehension of interview questions. Questions were also guided by our study objectives, which were to (i) characterize the unique features of rural areas as they relate to substance use, health and well-being, and access to health and social services; (ii) understand substance use and service access histories and motivations for enrolling in the TiOAT programs; (iii) examine the impact of contextual factors on access to TiOAT programs; and (iv) assess program changes and individual risks, harms, and health outcomes across time. This article focuses specifically on the objectives related to rural contexts and TiOAT access, with subsequent articles focusing on the other objectives (e.g., motivations, outcomes). In the context of the TiOAT programs, we operationalize the word ‘access’ to refer to a means in which an individual is able to fully participate in their respective program by having the opportunity to acquire their maximum daily dose without any barriers. The interview guide covered an array of topics based on both the study objectives and feedback from our community advisory board, and included broader questions to capture participant day-to-day experiences (narrative inquiry) and specific questions related to the context of the intervention (case study) [28].

### Data analysis

Interviews were audio-recorded and transcribed by an external transcription service with identifying information removed to protect participant confidentiality. Participants were assigned participant numbers as identifiers (e.g., D7, K14). Research team members reviewed the interview transcripts and developed a coding framework for thematic analysis [33]. Specifically, GB and MM reviewed their interview notes and transcripts individually and then met to discuss the findings and potential themes to develop our coding framework. We took an interpretivist epistemological standpoint to understand participants’ individual experiences and shared social meanings across the interview data [29] with attention paid to contextual factors of both the intervention and the geographical area (case study) and understanding how participants’ stories offer insights (narrative inquiry) into TiOAT program access [28, 29]. Transcripts were organized and stored in NVivo12, where they were coded thematically based on our study objectives, existing literature, and themes identified by the research team [34]. Given the small number of participants at the Duncan site (*n* = 7), we were unable to identify any noticeable differences in the experiences across sites during the data analysis process. As a validation measure, and to establish trustworthiness [35, 36], preliminary findings were shared with our community advisory board as well as a stakeholder group comprised of public health authorities, governments, researchers, policymakers, and community organizations (including those operating TiOAT clinics). Involving multiple stakeholders at various stages of the research process is considered best practice in conducting trustworthy research [35, 36]. This study was approved by the University of British Columbia/Providence Health Care Research Ethics Board and Island Health Research Ethics Board.

Due to the small size of the programs as well as the rural/smaller urban communities, and to protect participants’ anonymity, we do not share sociodemographic characteristics (e.g., age, gender, race) after each quote. See Table 1 for sociodemographic characteristics across the sample.

**Table 1:** Sociodemographic characteristics (*N* = 32)

**Age**	
Average (mean)	43
Range	26–60
**Gender**	
Woman	11
Man	21
**Race/Ethnicity (All that apply)**	
Indigenous	14
White	23
Black	1
Middle Eastern	1
Undisclosed	3
**Housing**	
Supportive housing	10
Apartment	5
Shelter	10
Homeless/unsheltered	6
**Past 30 Day Drug Use**	
Cocaine (powder)	5
Crack cocaine (rock)	10
Crystal methamphetamine	29
Heroin (down)	10
Fentanyl (down)	32
Opioids (diverted pharmaceuticals)	6
Benzodiazepines	10
Alcohol	7
Cannabis	13
Other	9
**Preferred Drug**	
Crystal methamphetamine	7
Heroin (down)	5
Fentanyl (down)	21
Alcohol	2
Cannabis	1
Other	3
**Past 30 Days Modes of Drug Consumption**	
Inject	13
Smoke/Inhale	30
Snort	4
Ingest/Swallow	8
**Opioid Agonist Treatments**	
Methadone	18
Buprenorphine/naloxone	3
Morphine (slow- and immediate release)	5
Hydromorphone	27
Other	1
**Past 30 Days Income**	
Full-time employment	1
Part-time employment	4
Selling drugs	6
Sex work	1
Recycling	10
Panhandling	5
Reselling goods	2
Social assistance	26
Other	7

### Positionality

Researchers in narrative inquiry become co-creators in the meanings of participant stories and therefore need to be reflexive on positionality throughout the research process and how this plays a role in interviewing, data analysis, and interpretation [30]. GB is a cis-man and spent much of his life living in rural Canada and has many years’ experience working in health services for, and conducting qualitative interviews with, people who use drugs. Since 2019, MM (cis-woman) has received training in qualitative research methods and has worked on a variety of harm reduction-focused studies with people who use drugs. Our various lived experiences influenced our interviewing process and how we developed the coding framework. For example, given the lead author’s research and lived experience in rural settings, there may have been less probing around the specifics of the rural context. In terms of data coding and analyses, if the authors supported abstinence-only approaches to substance use, we likely would not have interpreted some themes as negative (e.g., medication interruptions). Some of the authors have lived experience using illegal drugs and all of the authors support low-barrier, stigma-free harm reduction programs that increase the autonomy and improve the health outcomes of people who use drugs. Undoubtedly, our positionalities shaped our understanding of participant stories and the interpretation of the findings presented herein [37]. Thus, in utilizing a collaborative and team-based approach, reflexive and open dialogue allowed study team members to share their assumptions and interpretations, as well as challenge others throughout the research process [38, 39].

## Results

TiOAT access varied considerably. Such factors affecting access include distance and location of clinics; dispensing practices (e.g., daily-witnessed ingestion, take-away doses); social environmental characteristics; and medication interruptions in hospital and custodial settings. Below, we elaborate on each of these factors and how they impacted TiOAT access.

### Distance and location

The location where participants were living played a vital role in their ability to access the TiOAT clinics. Due to the high cost of housing across both study sites, the majority of participants were homeless (*n* = 16) or living in supportive housing (*n* = 10). Those who were staying in a shelter described the relative ease in accessing the TiOAT program. As described by one participant:

*The shelter is pretty close to the methadone clinic and the pharmacies that run the TiOAT program and mental health … it’s all kind of in the general same area. Like so for people on the TiOAT program actually living at the shelter makes sense…I don’t think everyone on the program does, there’s probably a lot that don’t but I know there’s a lot that do live in shelters on this program.* (K1)

Staying at the shelter provided an added convenience of being in close proximity to health services in the same neighbourhood. However, not everyone who was homeless was staying in emergency shelters. Others slept in vehicles or tents, which varied in locations. For example:

*When, when we had stayed at our first campsite, which was near the river, it was a lot harder for us to access it because it was the other side of town. Being dope sick [i.e., experiencing withdrawal] and trying to get to the other side of town is not fun. So we moved closer to the site.* (D1)

Participants living in supportive housing on the Northshore described the relative ease in accessing the clinic there: *“The location’s good too. Everybody kind of sticks around this area of the Northshore”* (K16). However, for those living elsewhere, including in housing that is more affordable on the outskirts of town, access became more onerous. Public transportation is limited or nonexistent in some areas, so a shuttle bus service was provided to some TiOAT participants in the Kamloops area:

*[My housing] is about 10 minutes out of town. [Interviewer: How do you get there usually?] Shuttle bus. They provide a free bus. It’s not a good situation, but it’s cheap rent.* (K2)

*Where I was living, it was really close, but now where I’m at now, it’s a little bit harder to get to… it’s kind of out in the middle of nowhere kind of thing and we can only get a ride in twice a day, so it is a little bit harder to access and to get to, and then go back home…There is no bus service out where I live… For us to catch the shuttle, it is either at 9 in the morning or 3 in the afternoon, and that’s it. [I: if you get on the 9 am shuttle, you have to wait around all day until you can go back?] Pretty much, yeah. [I: Does that impact your decision if you’re going to come into town or not?] Oh yeah, yeah.* (K4)

A lack of reliable and frequent transportation was a barrier to TiOAT access for those with housing that was in close geographic proximity to the program sites. No shuttle bus service was reported by participants of the Duncan site, demonstrating even more of an access challenge. For example:

*I live kinda in the bushed area. If I were to walk it takes probably about an hour and a quarter…I try to receive a ride from [someone] to at least get dropped off into town to walk the rest of the way.* (D5)

The above excerpts demonstrate how proximity and transportation play prominent roles in one’s level of access to TiOAT programs in rural and smaller urban settings.

### Dispensing practices

Similar to other OAT programs in BC, the TiOAT programs across both study sites required participants to attend the clinic multiple times a day for their daily witnessed ingestion (DWI), which was usually supervised by nursing staff. There were mixed feelings among study participants regarding such dosing schedules that required multiple daily visits to the clinic. Some participants spoke about how they liked having a daily routine and that the TiOAT program provided some structure. For example: *“It works for me. I don’t mind coming every day; it gives me something to do. It’s only a few blocks [away]”* (K3). However, the majority of participants described how having to attend the clinic multiple times per day impeded their abilities to do anything else (e.g., *“you can’t go anywhere or do anything”* [K14]), and how DWI would impact their employment opportunities:

*The fact that I have to spend the whole day basically at the location ‘cause you got to take it and hang out for 15 minutes and then another 45 minutes down the road you can have some more. Like so it kind of ties you to the building, right? It doesn’t give you much freedom to do anything else…If I was trying to do a construction job or something like that it wouldn’t work out too well, would it?* (D3)

In addition to the limits placed on participants via DWI policies, participants from Kamloops specifically reported other challenges with the TiOAT program, as medications were only accessible during the daytime hours of operation: *“From 5 o’clock at night ‘til 9 in the morning you’re SOL [shit out of luck] sort of deal”* (K14). While take-away doses (i.e., *“Carries”*) were preferred by many participants, those without them had to rely on using illegal drugs outside of clinical hours: *“at least [with carries] I would have stuff instead of using street drugs in the evening”* (K18). Only participants at the Duncan site were provided take-away doses in the evening: *“they give you carries at the end of the day…they’ll let you leave with 6 tablets”* (D3). Some participants in Kamloops reported that carries are only available to those who are able to provide an illicit opioid-negative urine drug sample, which would be challenging for those who procure illicit opioids to address withdrawal outside of clinical hours.

### Relationships and sociability

While participants described the challenges with DWI policies, their experiences in the TiOAT clinical settings were largely positive. Almost all participants described positive therapeutic relationships with clinical staff (nurses, pharmacists). These interactions provided them with the ability to have a role in dosing decisions as well as providing linkages to other health and social services (e.g., housing), leading to improvements in overall health and wellbeing (as we described elsewhere). The relationship between program participants and clinical staff were describe as creating social and familial bonds. For example:

*It’s way different. I mean, yeah, the pharmacists were great where we got our methadone from, but they… treated us civilly like they would any other customer and, you know, that’s about as far as it would go. But here, you’re a little closer and interact a lot more, so it’s… I don’t know. We’ve come to call ourselves our TiOAT family.* (D1)

Pharmacy staff in Kamloops were discussed as going above and beyond their roles in providing TiOAT medications to participants as described in the following excerpt:

*Well the staff here are awesome. Like the staff at this particular pharmacy are amazing, like the pharmacists are all really like good, uh, they deal with some of the people in the world that are judged by humanity, right? They welcome them into their store and you know they have a tab system here so if we haven’t got paid, they let us take like a chocolate bar or pop and you know we can sit and they have a little section of the pharmacy blocked off for TiOAT patients…We can all sit there and chat. It’s really, they’re amazing here.* (K1)

Conversing with others was reported as an important cultural element of drug use and how this extended to the TiOAT program via staff and other program recipients led to more positive engagement, as illustrated in the following quotes:

*Well, I always thought drugs was a sociable thing, right? So, when I do get drugs, I like to find somebody to get high with. It has always been that way with me. The [staff] here at the TiOAT program, they all converse when I’m doing my drugs, so it is actually like they’re getting high with us but they’re not doing drugs. That is what it’s kind of like, you know what I mean, it is socializing.* (K3)

*So, I like coming [to the TiOAT clinic] and, you know, seeing everybody I know and you know it’s like Norm walking into the bar at Cheers, you know, everybody knows him and people say hi and yeah, I like the social aspect of it for sure.* (D4)

Sociability and a sense of community within the TiOAT program – among participants, nurses, staff, and pharmacists alike – was a unique experience for participants who had not had these same experiences in other OAT programs.

### Medication interruptions

Multiple participants described instances when their access to TiOAT medications was interrupted by factors outside of their control. Most notably, participants described issues with dosing continuity when they were in hospital and carceral settings. Participants reported experiencing withdrawal in hospitals due to an inability to access their TiOAT medications. For example:

*I was in the hospital for ten days and I ended up going through withdrawal because they wouldn’t give me my TiOAT there […] There is something wrong with this hospital…They should actually have the TiOAT program in the hospital. I also tried to get access to methadone at the hospital and they would not give it to me even though I was on a prescription and everything. I ended up going without.* (K3)

*I did end up in the hospital and it’s been horrible. Um, it’s, uh, I go through really bad withdrawal and every time I’ve gone to the hospital I haven’t got to access my TiOAT. Maybe it’s cause I don’t have carries. I don’t know. You think they would have allowed it in the hospital.* (K1)

Participants were dismayed by their inability to access TiOAT medications in a hospital setting where access to hydromorphone for patients should not be onerous. An inability to access prescriptions in these settings may lead individuals to access illicit drugs to address their withdrawal symptoms: *“I could have somebody bring me dope so I could be taken care of one way or another but at least the dillies [i.e., Dilaudid] are cleaner”* (D4).

This lack of access to TiOAT medications also occurred in carceral settings, where participants described a lack of options. For example: *“I’ve been to jail several times. If I was to get thrown in jail I wouldn’t get – I mean you know I could get methadone probably but I’d never get the Dilaudid and all that”* (D6). Another participant who had discontinued participation in the TiOAT program, reported how a lack of OAT options while incarcerated was the main reason for no longer being enrolled in the program:

*When I went into jail, I got kicked off and they said they were going to continue the TiOAT program in jail and I thought you know what, that’s pretty cool. I didn’t think they’d be giving Dilaudid out in jail…and they didn’t. They ended up cutting me off. And then I just didn’t ever go back on it. (K21)*

Participants also described similar experiences when temporarily in police custody. For example:

*I was arrested and told the guard that I was a part of this program and if we could call [program staff] to bring me meds. And all I was told was “I’ll talk to the main guard and see what happens.” Nobody came back and by the time I was released, it was too late to get a dose or my carries, so I was screwed for the day. [I: And so what did you do?] I had to take street drugs, otherwise I would have been in pain and puking and all that fun stuff.* (D1)

The above experiences illustrate how a lack of medication continuity across hospital and carceral settings can lead to program discontinuation as well as negative health outcomes (e.g., withdrawal, overdose risk).

## Discussion

In summary, our findings demonstrate that a variety of factors affect TiOAT program access in Duncan and Kamloops. Proximity and access to transportation, medication dispensing schedules and policies, staff relationships and social interactions, and program interruptions in hospital and custodial settings affected participation in the TiOAT program. Taken together, these findings highlight the experiences among study participants who live in rural and smaller settings and the need for policy and practice changes to improve TiOAT program access.

There is very limited research on OAT access in the rural Canadian context and the majority of existing studies rely on medical records and administrative data and are from the province of Ontario [40]. There is a lack of research in other Canadian provinces, including in BC, as well as minimal qualitative research that examines barriers and facilitators to OAT access [40], and to our knowledge no studies to date that have examined TiOAT programs in rural and smaller urban settings in Canada. Our study builds on existing literature (primarily from the US) that identifies transportation barriers to substance use care in rural settings [41–45]. For example, one study examining rural versus urban differences across 5 US states reported significant disparities in travel times, where in rural areas it was 6.3 times greater than in large metropolitan cities [42], demonstrating the challenges of accessing care among those who do not live in close proximity to clinics. This was an issue for our study participants who lived in housing that was more affordable and located on the peripheries of their respective communities, but who then had to rely on infrequent shuttle bus services, or worse, had to walk long distances if there was no available public transportation.

Similar to our findings on DWI policies, research on TiOAT access in Vancouver, BC, also reports DWI as a barrier to program engagement [25] and similar findings have been described in large urban settings related to access to traditional OAT programs such as methadone [46, 47]. This program barrier in our study setting is further compounded with rural-specific transportation and proximity issues (as described above) that create further challenges with attending a clinic daily. DWI created employment access issues due to requirements to attend the clinic multiple times daily to receive their medications. Unsurprisingly, research reports a negative association between people enrolled in methadone programs and employment initiation [48]. There is therefore a need for more flexible policies in our study settings that provide individuals with take-home doses. Aside from providing participants with more autonomy and opportunities for employment, it would also reduce their overdose risk, particularly for participants who rely on using toxic illicit drugs in the evenings to address withdrawal and pain. Easing regulations around opioid dispensing and take home doses may lead to concerns around diversion and community safety [49, 50]. However, studies have reported how loosening opioid prescription regulations during the COVID-19 pandemic has had positive effects on treatment retention and has not led to substantial increases in adverse events (e.g., illicit opioid exposure, overdose) [51, 52]. Furthermore, BC coronial data indicates that very few overdose deaths occur from using prescription opioids (2% of cases from 2015–2017; the majority are the result of the use of unregulated fentanyl) [53] and use of diverted prescription opioids has been found to have protective benefits [54–57], with one recent study reporting the use of diverted opioids being associated with reduced risk of unregulated fentanyl exposure [57]. However, additional research is needed, including in rural and remote communities, to understand any risks and benefits not captured herein. To minimize any other perceived risks, communities should also explore medication delivery options to alleviate travel constraints, particularly in jurisdictions where there is a lack of reliable public transportation.

That participants described positive therapeutic relationships with TiOAT program staff and clinicians, and how these relationships created familial and social bonds, may demonstrate a strength to the programs in Duncan and Kamloops. These findings illuminate experiences that contrast to the surveillance and stigma experienced by people who use drugs in other healthcare settings [58–61], as well as in rural BC more generally [62]. Existing OAT research on the provider-patient dyad in the rural Canadian context, highlights what Pijl et al. describe as a “narrow biomedical focus” and there is a need for research to measure successes that encompass more of a holistic understanding of healthcare access that includes “the importance of community support, culture, social restoration, and belonging” [40]. Our findings demonstrate experiences of support and familial belonging as well as how the sociability of drug use translated to the TiOAT clinical setting. Past research has highlighted the importance of sociability as an engagement and harm reduction strategy among people who use drugs in health service settings [63, 64], as well as the need for public health interventions, including safer opioid supply programs, to not just address individual-level harms, but also overarching social and structural contexts [65, 66]. The TiOAT programs in both Duncan and Kamloops not only provide a service intended to reduce the harms of illicit opioid use, but also a refuge where participants felt socially connected to both staff and their peers. It is important to consider if these positive relationships may be attenuated if people did not have to undertake DWI. These bonds may not be the result of DWI but rather attributed to staff who actively create a non-stigmatizing organization culture and participants could arguably still feel socially connected while attending a clinic less frequently – though additional research is needed to understand how these social and organizational factors may lead to optimal delivery models. Ultimately, participants should be provided a choice on medication dispensation to alleviate any access barriers while also providing opportunities for social connection.

Lastly, in custodial and hospital settings, participants described an inability to access their TiOAT medications, leading to negative health outcomes. Unlike larger urban settings (e.g., Vancouver) where hospitals are more adequately equipped with substance use health services, harm reduction programming, and addictions specialists [67], this is less the case for hospitals in rural communities making continuity of receiving novel OAT medications a challenge (despite hospitals likely having a stock of hydromorphone to treat other conditions). Aside from experiencing opioid withdrawal symptoms, not being prescribed TiOAT in hospitals may lead to early discharge, overdose risk from using illicit drugs, as well as other related negative health outcomes. These dynamics illuminate a critical gap and a need for more comprehensive substance use care in rural hospital settings. In custodial settings in BC, methadone and buprenorphine are available in both provincial and federal correctional facilities [68] and utilization has more recently increased following provincial healthcare insurance coverage for buprenorphine [69]. Our findings demonstrate the need for TiOAT medications to be added as an available OAT option for individuals in carceral settings to prevent poor health outcomes and maintain a continuity of an individual’s TiOAT regimen both during and after incarceration. Past research among HIV-seropositive people who inject drugs demonstrates a relationship between incarceration and nonadherence to antiretroviral therapy and calls for the delivery of substance use care for people who use drugs in prisons [70, 71]. Further, a recent study in BC indicates that from 2015 to 2018, only 25% of individuals with an opioid use disorder were dispensed OAT within two days post-release [72]. It is well-documented that people released from prisons are at an increased risk of overdose mortality [72–78]. Additionally, OAT retention is associated with a reduced risk for overdose death [79, 80]. As such, novel public health interventions, including TiOAT, are urgently needed to prevent overdose and address other substance use-related harms among individuals recently released from carceral settings.

There are some limitations to this study. This dataset is from baseline interviews and some participants had been enrolled for only 1-month, so there may be other factors over time that we were not able to capture. Follow-up interviews are therefore needed in order to capture any changes over time including additional factors affecting program access. Aside from the take-away doses policy differences across the programs, we were unable to ascertain differences in experiences of access across the sites due to a small number of participants at the Duncan site. Future research should examine differences based on levels of rurality. Also, while we reached a diversity of participants across the two programs in terms of sociodemographic characteristics (see Table 1), the results reported herein may not be representative of all participants in TiOAT programs in our study settings nor in other rural jurisdictions. Finally, the average age of participants was 43 and these results therefore may be less relevant to youth enrolled in similar programs. Additional research, including studies guided by implementation science [81], is needed to understand differences across various demographic characteristics in rural settings, including age, Indigeneity, and gender and how these may impact implementation and access to TiOAT programs.

## Conclusions

To conclude, this was the first study on TiOAT programs in rural and smaller urban settings in Canada. Our findings highlight the unique challenges faced by TiOAT program participants in rural and smaller urban settings in BC. They also demonstrate the beneficial ways in which health services targeted toward people who use drugs can operate by creating an environment free of stigma and with an emphasis on social bonds and belonging. Public health authorities in rural and smaller settings should consider these factors when designing, implementing, and scaling up future substance use services.

## Data Availability

The qualitative datasets for this study are not publicly available given the sensitive nature of the topic, including confidential information that could compromise participant confidentiality and consent.

## Abbreviations

BC: British Columbia
COVID-19: Coronavirus Disease 2019
OAT: Opioid agonist therapy
TiOAT: Tablet injectable opioid agonist therapy
